# The Work of the Holy Ghost

**Published:** 1887-07-30

**Authors:** 


### Words of Consolation.

[Communications for this column should be addressed, "Chaplain," care of the Editor of The Hospital, who will gratefully welcome any suggestions or inquiries from matrons, nurses, and the staff generally.]

XLIII.—The Work of the Holy Ghost.

"He will show you things to come" is as true of the Comforter as it is of the Guide. He is a very present help in trouble, in that He points so clearly to the sunshine beyond the cloud. The soul, therefore, by discerning the light to come, sees also that there is a vast difference between the passing cloud and the darkness of the night. You want a good deal of light sometimes in order to discern the size and shape and the thickness of a cloud. A strong light thrown upon a cloud, black as it may make the cloud look, tells you plainly that the dark mass is but a vapour which must soon pass away. In this way many a suffering disciple is led by the comfort of the Holy Spirit to discern the meaning and the ends of the shadow cast over his life. The Spirit helps him to see the due proportions of the cloud, taking away by the light which He supplies all the unfair reasoning, and magnifying of grievances, once so easily given way to. And when the due proportions and purpose of the cloud are recognised, then, and not till then, signs are manifested of the glory beyond. We have already pointed out how especially true this appears to be in the case of dying Christians. When the heaviest cloud of all is overhanging, how exactly do they seem to have its true proportions revealed to them; how soon do they discern the edges all gilded with light. Yet to others the appearance is changed; the cloud has no limit, the darkness is of the night, for "the world cannot receive Him." In all time of our tribulation, and in the hour of death, there is certainly no better testimony to the truth of the Christian religion than the presence of the Comforter; but this is a testimony (like all others given by our Lord) far more easily perceived and accepted by those who are previously disposed to faith. We cannot yet see beyond the hour of death: nevertheless we may rest assured that the office of the Comforter will be continued until the day of Judgment. "In that solemn hour" (says Newman) "we shall have, if we be His, the inward support of His Spirit carrying us on toward Him, and witnessing with our spirit that we are the children of God. God is mysteriously threefold: and while He remains in the highest heaven, He comes to judge the world; and while He judges the world He is in us also, bearing us up, and going forth in us to meet Himself. God the Son is without, but God the Spirit is within; and when the Son asks, the Spirit will answer. That Spirit is vouchsafed to us here; and if we yield ourselves to His gracious influences, so that He draws up our thoughts and wills to heavenly things, and becomes one with us, He will assuredly be still in us and give us confidence at the Day of Judgment. Gifted with that supernatural strength we may be able to lift up our eyes to our Judge when He looks upon us, and look on Him in turn, though with deep awe, yet without confusion of face, as if in the consciousness of innocence."

(*To be continued.*)

